# Best practices in global health evaluation: Reflections on learning from an independent program analysis in Bihar, India

**DOI:** 10.7189/jogh.10.020395

**Published:** 2020-12

**Authors:** Kala M Mehta, Victoria C Ward, Gary L Darmstadt

**Affiliations:** 1 Department of Pediatrics, Stanford University School of Medicine, Stanford, California, USA; 2 Department of Epidemiology and Biostatistics, University of California San Francisco, San Francisco, California, USA

Frameworks and guidelines are commonly used by public health practitioners and medical researchers to improve research quality and to guide program assessments and reporting [1-6]. Best practice recommendations have been suggested for a number of topics in low-and middle-income country (LMIC) contexts [7,8] and several calls for a common set of best practices for collection and utilisation of large, complex health-related data have been issued [9,10].

Here we reflect on lessons learned from our three-year independent synthesis of learning from *Ananya*, a complex primary health care program funded by the Bill and Melinda Gates Foundation (BMGF) and implemented by the Government of Bihar (GoB) with ancillary support from multiple civil society and academic partners, aimed to improve reproductive, maternal, newborn, and child health and nutrition (RMNCHN) statewide in Bihar, India [11]. We describe the steps and processes by which our multidisciplinary, cross-national team collaborated to acquire and analyse data, and report findings from the *Ananya* program with an aim to inform the efficient and effective use of complex secondary data for independent program evaluations in LMIC contexts.

## FORMING A PARTNERSHIP NETWORK AND BUILDING TRUST

Capturing program learning required working with several organisations, which was dependent on building trust within the partnership network. Communication from the funder to the partners regarding the role of the evaluator and expectations regarding provision of documents and data by the partners can be very helpful in this process. Given the sensitive nature of data sharing and evaluation, the funder can also play an active role early in the process as an independent motivator and facilitator of shared goals of the partnership.

A key initial step was to define priority topic areas, including hypthoses to be tested. Key individuals were identified from all partners and included policy makers, program designers, implementers, evaluators and disseminators. A forum was established for regular open dialogue of the partnership network and was critical to success. Governance, structure and communications for the partnership were discussed. In retrospect, however, our partnership would have benefitted from further definition of roles and accountabilities with respect to use of data and reporting of findings. Agreements were needed on processes for making fully informed decisions about elements in the evaluation, such as the influence of contextual factors and choice of indicators, in a way that maintained the independence of the evaluation without eroding the essence of partnership.

## UNDERSTANDING PROGRAM BACKGROUND AND CONTEXT

In order to understand the heterogenous program and historical context of *Ananya*, we undertook an extensive review of relevant documents describing the ‘pre-context’. Use of PRISMA guidelines can help to promote a shared understanding of review processes and requirements [2]. We reviewed hundreds of program documents and gathered publicly-available data sources external to the evaluation, including the Annual Health Surveys and National Family Health Surveys, for triangulation purposes at a later stage. Extensive communications, including key informant interviews, were held with each of the partners to understand the nuanced history of implementation, including barriers to success. Conference proceedings, presentation materials and audio recordings of meetings from all partners were reviewed to understand the perspectives and lens through which the data had been interpreted and presented. We made multiple trips to India to formulate partnership agreements and acquire data through data sharing agreements, and to further discuss details of the data. We additionally undertook Group Model Building as a means of developing a shared view of inter-relationships among various program components [12]. The result was a depiction of the social, economic and political context in which the program took place, a consolidated Theory of Change, a project timeline of implementation, and improved understanding of evaluation study designs.

A mutually agreed upon shared mental model can be helpful in ensuring the buy-in and collaboration of all partners. This agreement may be achieved through a common communication platform and a shared document library with pertinent literature to inform the knowledge network, made accessible to the entire team. Ensuring that a process is established for document contribution, sources of information, and multi-stakeholder review aids efficiency.

## UNDERSTANDING DATA SOURCES IN THE CONTEXT OF IMPLEMENTATION

A consolidated timeline of interventions and data collection across program partners was developed. The contents of each data set were mapped to determine which data should be used to evaluate which intervention and in what timeframe. This required transparent sharing of data and corresponding files as well as a collaborative review of data, including data quality. Knowledge of external drivers was necessary to understand what may have advanced or limited subsequent outcomes. Variation in the frequency and strength of interventions may provide a unique opportunity to study what we term ‘intervention dose’; however, this is only possible if information about intensity, time and place of intervention is captured and documented. Documentation of changes in the external environment that may affect intervention dose are also important.

## DATA ASSESSMENT AND INDICATOR SELECTION

Ideally, research questions and methodological approaches – including indicator selection – are defined pre-intervention by all members of the partnership network to ensure that answers derived will tie to specific, measurable programmatic changes for the stakeholders. This is similar or parallel to community-based participatory research or implementation science principles [13,14]. However, the process of building consensus on what should be measured and by whom is still fraught with challenges [15]. Research questions may span hypothesis testing and hypothesis generation, and should specify predictors, outcomes, study population, potential sources of bias, and mitigation strategies. Following the identification of research questions, a detailed study protocol should be developed, which can be sent out for review to the entire research network for feedback, and ideally also for peer review as a protocol publication.

**Figure:**
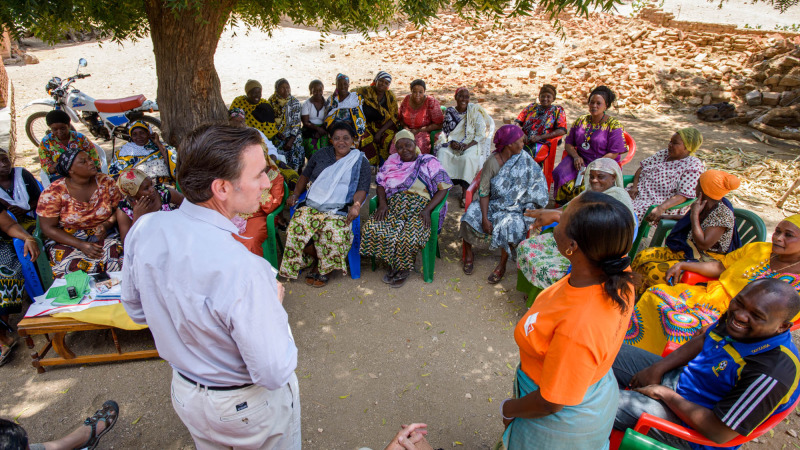
Photo: From the Bill & Melinda Gates Foundation, photographer Barbara Kinney, used with permission.

In the case of *Ananya*, surveys were implemented by different partners with various areas of focus (eg, frontline worker platform, facility-based quality of care, communications, self-help groups). In choosing indicators across these surveys after they had been completed, we sought to identify a common ‘minimum set’ of questions that were consistent, including identical wording of the stem question, the skip pattern in the survey, as well as the answer choices. We sought to apply principles for good practice in the reporting and conduct of survey research [16], roughly following the MOOSE guidelines for reporting observational studies [3]. Indicator selection and assessment should ideally enable comparisons between data sources as well as within data sources (eg, serial rounds of a given survey). Each specific survey may have additional items to understand the specific contribution of that particular intervention or time period. Given that the tenants of an external evaluation should ensure that indicators are chosen independently to minimise bias, the external Stanford team took responsibility for indicator selection. Data repositories across data sets were harmonised with consistent, carefully documented definitions. Raw data sets were retained in unaltered form, and all changes to the data in the process of cleaning and harmonization were documented. We selected indicators prior to analysis which were linked to programmatic focus and articulated goals, and representative of the health of beneficiaries and potential contribution to policy decisions. Final indicators chosen were discussed with program partners to gather further input on their relation to program implementation. In addition to thorough review internal to the Stanford team, a series of meetings were held with members from CARE India’s Concurrent Measurement and Learning team to review each indicator used in their Community-based Household Surveys (CHS). This ensured identification of a context-relevant set of indicators and documentation of how we calculated each indicator.

## PROTOCOL DEVELOPMENT AND STATISTICAL ANALYSIS PLAN

Protocols were written including a statistical analysis plan (SAP) that pre-specified the details of evaluation methods [17]. This is particularly important for studies with complex survey design. All stakeholders should agree with the SAP before analysis begins. Power analysis and how to handle missing data, sensitivity analyses and subgroup analyses should be prespecified.

## DATA ANALYSIS

In *Ananya*, we sought to optimise use of secondary data, including recalculation of the study weights of the CHS, given that the data were collected using a methodology that varied for the two intervention phases [11]. Our recalculation of the weights ensured that we were able to compare estimates spanning 2012-2017 using equivalent methods despite design differences.

Another challenge we encountered was obtaining differing estimates across seemingly similar indicators of various *Ananya* evaluations. This required our team to determine which data set and indicators were most reliable for a specific purpose. We found, for example, that results on immunisations were different in Mathematica vs CHS data, even though indicators and timeframe were roughly similar. We shared these comparative analyses with the implementors, and together agreed that variation can exist, due, for example, to minor differences in questions and possibly due to differences in training and supervision of data collectors.

## DOCUMENTATION AND DISSEMINATION OF RESEARCH

Decisions regarding authorship should be discussed by all contributors at the beginning to ensure alignment in incentives and expectations and adherence to International Committee of Medical Journal Editors criteria. Recognition of the implementing partners’ investments and efforts is critical, with careful consideration of authors from global south and global north countries. An additional issue to consider is the inclusion of members of the funding body in evaluation authorship. Some peer-review journals will not consider an analysis as an “independent evaluation” should funders be included as authors. Thus, clear policy by the funder at the time of contractual engagement should be agreed upon, recognising the potential impact on the journals which may be considered for publication. Ideally the partner network is involved in the decision of whether the evaluation is better suited for publication without the funding partners, or whether involvement of the funders is necessary to strengthen trust among the partners. Formation of a Study Group, inclusive of all partners in the defined research network, may also enable wider recognition of contributions to the research.

Recent efforts to create within-country data repositories and rallies to aid in community-based participatory generation of research questions and team formation can be helpful in addressing issues of inclusion. Inviting patients and program beneficiaries to data rallies further advances inclusion and community relevance [18].

Manuscripts were disseminated to the full research collaborative for review during a meeting with all members. Careful attention was required to utilise input to improve accuracy and relevance of reporting while guarding against the introduction of bias. Manuscripts were then submitted for journal peer-review.

To facilitate use of the findings and lessons learned to inform policy discussion, results were presented to officials from the Government of Bihar, the Government of India and all key stakeholders. For any large-scale, complex evaluation to be successful, government review of the results and commitment to fund implementation of effective interventions is critical for sustainability.

## CONCLUSION

This manuscript presents lessons learned and proposed best practices in global health evaluations of complex programs, which may be useful to a wide audience of researchers, health policy experts and funders. Few prior documents have covered this topic. The closest may be the SUCEED [19], which is recent and does not take into account several levels of contextual information. Identified best practices may be helpful in guiding future global health evaluations to advance programmatic efficiency and performance improvement worldwide.
